# Performance of three colposcopic images for the identification of squamous and glandular cervical precursor neoplasias

**DOI:** 10.1007/s00404-021-06284-4

**Published:** 2021-11-02

**Authors:** Giselle Fachetti-Machado, Rosane Ribeiro Figueiredo-Alves, Marise Amaral Rebouças Moreira

**Affiliations:** Post-graduate Program in Health Sciences, School of Medicine, Universidade Federal de Goiás, Av. T4, esq. com T13, 1478, Salas 91B e 92B, Setor Bueno, Goiânia, GO 74230-030 Brazil

**Keywords:** Glandular and epithelial neoplasia, Cervical intraepithelial neoplasia, High-grade squamous intraepithelial neoplasia, Adenocarcinoma in situ, Diagnosis, Colposcopy

## Abstract

**Purpose:**

To evaluate prevalence and diagnostic performance of three colposcopic images to diagnose squamous and glandular cervical precursor neoplasias.

**Methods:**

Cross-sectional study, conducted through analysis of stored digital colposcopic images. To evaluate the diagnostic performance of three images, herein named grouped glands, aceto-white villi, and atypical vessels, for detection of adenocarcinoma in situ (AIS) and cervical squamous intraepithelial neoplasias (CIN) grades 2 and 3, calculations of sensitivity, specificity, accuracy, positive likelihood ratio, receiver operating characteristic (ROC) curve, and area under the curve (AUC) were made, with their respective 95% confidence intervals.

**Results:**

Grouped glands, aceto-white villi, and atypical vessels images had: prevalence of 21.3, 53.8, and 33.8% in patients with AIS, and 16.2, 19.5, and 9.3% in those with CIN 2 and 3; for the diagnosis of AIS, sensitivity of 21.3, 53.8, and 33.8%, specificity of 89.8, 95.2, and 94.9%, accuracy of 76.6, 87.2, and 83.1%, positive likelihood ratio of 2.1, 11.2, and 6.6, and AUC of 0.55, 0.74, and 0.64; for the diagnosis of CIN 2 and 3, sensitivity of 16.2, 19.5, and 9.3%, specificity of 89.8, 95.2, and 94.9%, accuracy of 39.4, 43.4, and 36.3%, positive likelihood ratio of 1.6, 4.1, and 1, 8, and AUC of 0.53, 0.57, and 0.52, respectively.

**Conclusion:**

Prevalence and accuracy of the three images were higher for the diagnosis of glandular than squamous cervical precursor neoplasias. Sensitivity, specificity, positive likelihood, and AUC of aceto-white villi and atypical vessels images were higher for the diagnosis of glandular than squamous cervical precursor neoplasias.

## Introduction

Invasive cervical cancer is the fourth most diagnosed type of cancer in women worldwide, and 90% of these tumors are carcinomas, malignant neoplasms of epithelial origin. Cervical squamous cell carcinomas account for about 65% of the cases, while glandular cell carcinomas, including several subtypes of adenocarcinomas, account for approximately 29% of them [1].

Simultaneously to the decline in the overall incidence of invasive cervical cancer, observed between the 1960s and the 1990s, an increase was detected in the absolute and relative incidences of different adenocarcinoma subtypes. This trend continued until the 2000s, when the incidence of invasive adenocarcinomas had a decline [2].

This period of increasing incidence of invasive adenocarcinomas must have been a consequence of women’s greater exposure to the risk factors for these types of cancer, especially persistent human papillomavirus (HPV) 16 infection associated with the relative inefficiency of cytology and colposcopy for the diagnosis of adenocarcinoma in situ (AIS) [2], consensually recognized as the precursor of invasive adenocarcinomas [3]. The identification of HPV as an oncogenic agent [4], continuous improvement in cytology, most uniform and reliable records of cytological and histopathological diagnoses, and adoption of new DNA detection technologies for the different types of high-risk HPV must have conjointly contributed to an increase in the detection of AIS in young women [5] and a decrease in the incidence of invasive adenocarcinoma observed since the beginning of the twenty-first century [2].

Colposcopy is indicated for women with cytological abnormalities and high-risk HPV infection, or HPV-16 and HPV-18 infection, when cytology results are negative or cannot be performed on the same sample already collected [6]. This procedure mainly aims to identify the most abnormal area in the cervical epithelium that should be biopsied. The histopathological diagnosis of the fragment obtained defines the selection of the ideal therapeutic method [7]. Despite this fundamental role in the invasive cervical cancer screening system, colposcopy involves a certain degree of subjectivity in the interpretation of images, which can lead to underestimated histopathological diagnoses [8].

The colposcopy terminology defined by the International Federation of Cervical Pathology and Colposcopy (IFCPC) does not include parameters related to glandular cervical neoplasias [9]. However, in 1999, a series of images were reported that could be associated with glandular cervical neoplasias, although they were similar to a normal transformation zone (TZ). Moreover, it is important to take into consideration that glandular neoplasms may be invisible to the colposcopist, since part of them can occur in the proximal portion of the endocervical canal and in the depth of its crypts [10].

The implementation of primary prevention of invasive cervical carcinomas using vaccination, associated with the progressive adoption of highly sensitive screening systems, implies earlier detection of cervical precursor neoplasias [11]. This scenario requires more efficient colposcopic procedures for the recognition of discrete images. Thus, these three patterns of colposcopic images, which resemble the images previously described by Wright [10], were identified in stored digital colposcopic images and studied for their prevalence and diagnostic performance regarding intraepithelial cervical, squamous, or glandular neoplasias.

## Methods

The study was approved by the Institutional Review Board and Ethics Committee of the Clinical Hospital, Universidade Federal de Goiás (CAAE: 03,421,418.8.0000.5078), conducted according to the Helsinki Declaration, and no signed written consent was required, since only stored digital images, medical records, and colposcopy reports were accessed.

This retrospective cross-sectional study was conducted between 2005 and 2018, in a private colposcopy service. Two colposcopists reviewed stored digital colposcopic images containing 640 × 456 or 720 × 480 pixels. Data collected from digitized standardized medical records and stored digital images, in LPT4 (LPT4 information systems, Curitiba, PR, Brazil) and Zscan (Zscan Software, 2001–2016, Goiânia, GO, Brazil) programs were age, parity, referral cytology, visualization of the squamous-columnar junction (SCJ), degree of colposcopic findings, and histopathological diagnosis.

Stored digital images of patients presenting with cervical precursor neoplasias diagnosed after analysis of excision specimens were included. Additionally, a random sample of stored digital images of patients that underwent cervical biopsy between 2005 and 2018 and did not present with abnormal colposcopic or histopathological findings was included. Digital image files with insufficient quality for reading and those in which SCJ was not visible in the initial colposcopic exam were excluded.

A single colposcopist performed the initial exams using D.F. Vasconcelos (Valença, RJ, Brazil) and Medpej Equipamentos Médicos (Ribeirão Preto, SP, Brazil) devices with five levels of enlargement (6×, 10×, 16×, 25×, and 40×) applying 5 or 10% acetic acid solutions and Schiller’s solution. Guided biopsies were taken with Gaylor-Medina forceps. All the stored images were reviewed by the initial examiner and a second colposcopist. Disagreements between examiners were discussed aiming to reach a consensus.

The cytological abnormalities were categorized following the Bethesda Cytological Classification, updated in 2014 [12]. The colposcopic findings were categorized as normal, minor findings, major findings, or suspicious for invasion, according to the terminology proposed by the IFCPC [9].

**Fig. 1:**
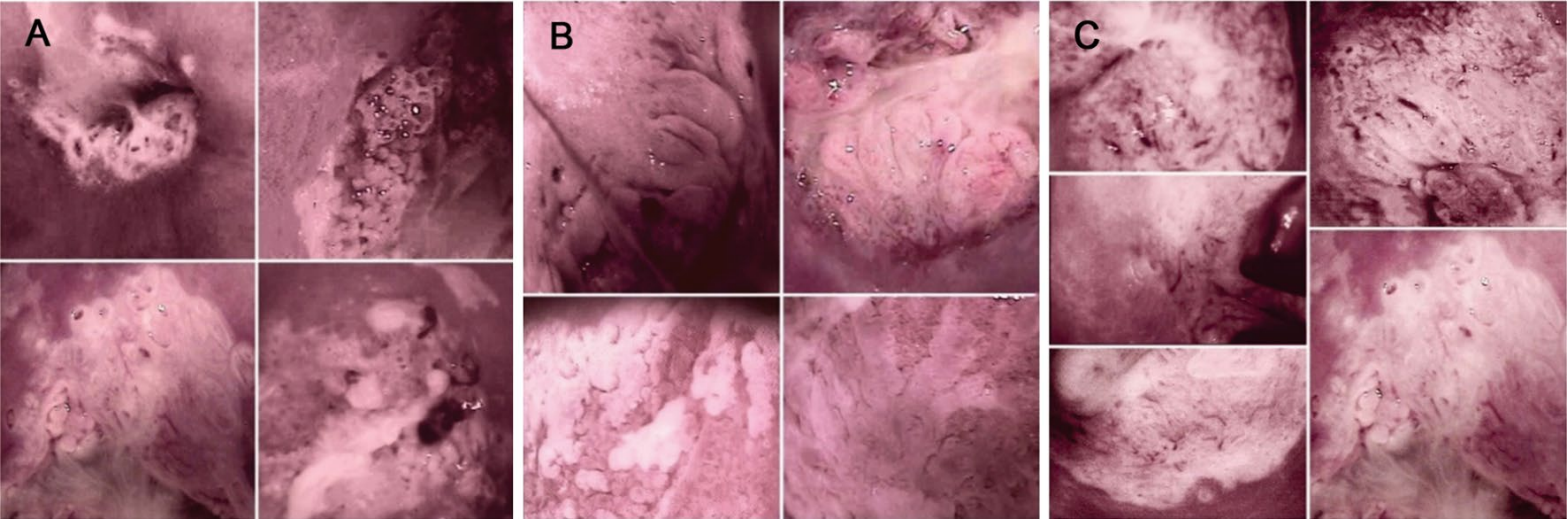
Three colposcopic images investigated in stored digital images. **a** Obstructed dilated grouped glands (grouped glands); **b** Aceto-white villi with invaginated borders fused or not (aceto-white villi); **c** Atypical vessels in cylindrical epithelium area (atypical vessels)

Three types of images were investigated in the digital files: obstructed dilated grouped glands, aceto-white villi with invaginated borders fused or not, and atypical vessels in cylindrical epithelium area, herein respectively named grouped glands, aceto-white villi, and atypical vessels. They were considered present only if identified in a TZ classified as a major colposcopic finding [9] (Fig. 1).

The TZ excisions were performed under local anesthesia and colposcopic vision using a Wavetronic 5000 Digital Hf Surgical Unit (Loktal Medical Electronics Ind. Com. Ltda, São Paulo, SP, Brazil). The histopathological exams of biopsy fragments and conization pieces were performed by a single examiner and classified following the World Health Organization International Tumors Classification [13] and Richart Classification for cervical intraepithelial neoplasias [14]. An Excel 2013 spreadsheet (Microsoft Corporation Redmond, WA, USA) was used for collected data entry, and an increasing identification number was generated for each participant.

### Statistical analyses

The collected data were analyzed using the Statistical Package for Social Sciences (SPSS) program for windows 21.0. Descriptive and frequency analyses of the three colposcopic images were performed according to the histopathological degrees and types of cervical precursor neoplasias.

The diagnostic performance of the three colposcopic images was evaluated by analysis of sensitivity, specificity, accuracy, and positive likelihood ratio (LR +) with their respective 95% confidence intervals (95% CI). The results of histopathological exams compatible with cervical squamous intraepithelial neoplasias (CIN) grade 1 or with no atypia were considered “absence of disease” (≤ CIN 1) and those compatible with CIN grades 2 and 3 or AIS were considered “presence of disease”.

The variations in sensitivity and specificity for each of the three colposcopic images were estimated by building two graphs with the receiver operating characteristic (ROC) curves and evaluated by calculating the area under the curves (AUC), considering the diagnostic prediction of glandular cervical precursor neoplasias in the first graph, and the prediction of squamous cervical precursor neoplasias in the second one. AUC values between 0.50 and 0.60 were considered fail, between 0.60 and 0.70 poor, between 0.70 and 0.80 fair, between 0.80 and 0.90 good, and between 0.90 and 1 excellent regarding diagnostic performance [15].

## Results

**Fig. 2:**
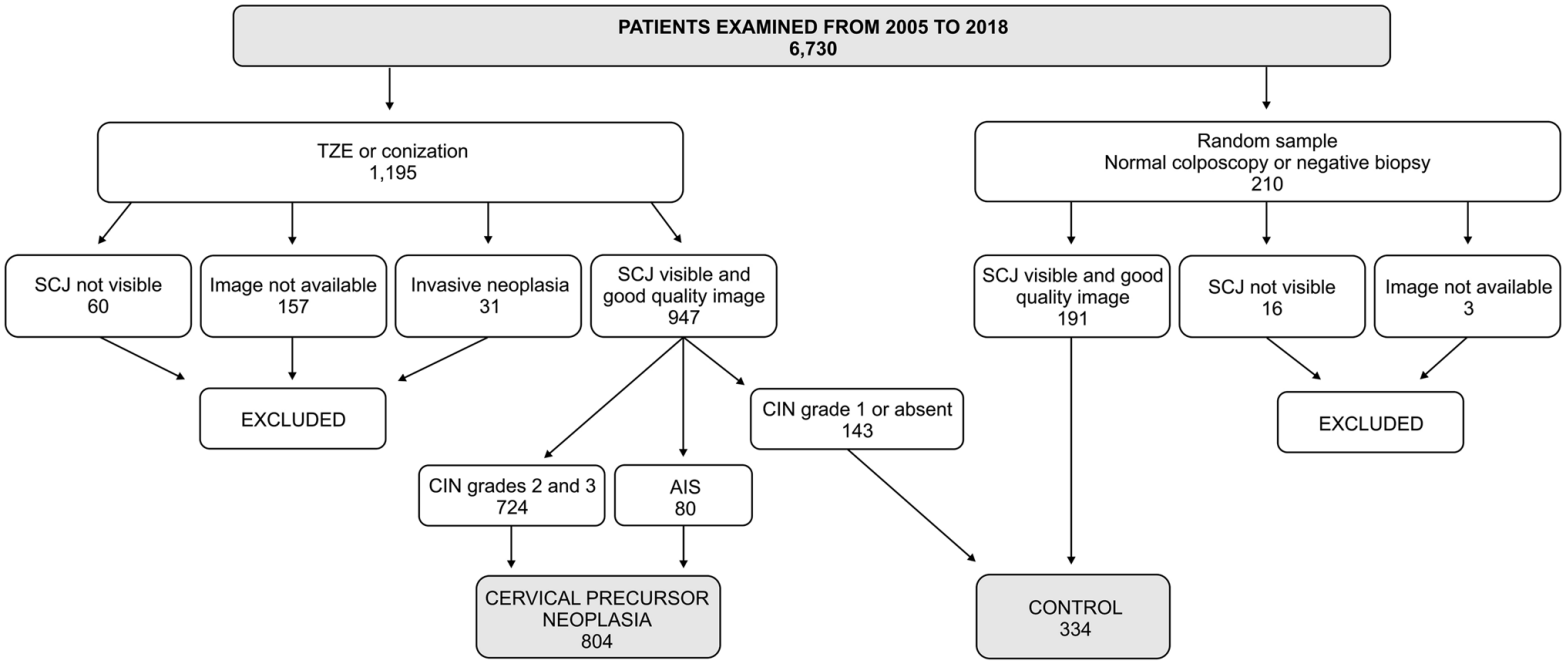
Flow chart of the sample

A total of 1138 patients that underwent colposcopy in a private service from 2005 and 2018 were enrolled in this study after applying the inclusion and exclusion criteria, 724 diagnosed with CIN 2 and 3, 80 with AIS, and 334 control (≤ CIN 1 findings with visible SCJ and good quality image) (Fig. 2). Two colposcopists evaluated a total of 12,436 digital photographs, an average of 10.9 images per participant. Of these, the digital images of 97 patients (8.5%) were considered low quality, but still possible to interpret (Table 1).

**Table 1:** Social and demographic profile, cytological, colposcopic, and histopathological findings in 1138 participants

Variable								
Final diagnosis	Control (≤ CIN 1)		CIN 2 and 3		AIS		Total	
	334 (29.3%)		724 (63.6%)		80 (7.0%)		1138 (100%)	
Age, years								
Range	20–49		15–73		17–66		15–73	
Mean (sd)	31.0 (24.6–37.4)		30.4 (22.8–38.0)		32.2 (22.5–41.9)		31.0 (22.7–39.3)	
Lifetime sexual partner^a^	*n*	%	*n*	%	*n*	%	*n*	%
≤ 2	125	37.4	187	25.8	31	38.8	343	30.1
> 2	190	56.9	463	64.0	37	46.3	690	60.6
Contraceptive method^b^								
Hormonal	156	46.7	405	55.9	52	65.0	613	53.9
Condom	8	2.39	26	3.6	3	3.8	37	3.3
None and others	92	27.5	204		17		313	27.5
Full-term pregnancy^c^								
≤ 1	248	74.3	596	82.3	67	83.8	911	80.1
> 1	81	24.3	125		13	16.3	219	19.2
Tobacco use^d^								
Past and current smoker	18	5.4	78	10.8	0	0	96	8.4
Never smoker	300	89.8	597		70	87.5	967	85.0
Colposcopy referral								
ASC-US/LSIL	237	71.0	313	43.2	23	28.8	573	50.4
ASC-H/HSIL +	50	15.0	358	49.4	33	41.3	441	38.8
AGC/AIS +	7	2.1	12	1.7	22	27.5	41	3.6
Others	40		41	5.7	2	2.5	83	7.3
SCJ placement								
Endocervical canal	103	30.8	225	35.2	14	17.5	342	30.1
External orifice	157	47.0	377	52.1	47	58.8	581	51.1
Ectocervix	74	22.2	122	16.9	19	23.8	215	18.9
Colposcopy findings								
Normal	20	6.0	4	0.6	0	0	24	2.1
Minor	200	59.9	124	17.1	3	3.8	327	28.7
Major	114	34.1	596	82.3	75	93.8	785	68.7
Suspicious for invasion	0	0	0	0	2	2.5	2	0.2
Still image quality								
Barely readble	35	10.5	59	8.1	3	3.8	97	8.5
Readble	299	89.5	665	91.9	77	96.3	1041	91.5
Number of new images/patients								
0	274	82.0	453	62.6	14	17.5	741	65.1
1	53	15.9	225	31.1	48	60.0	326	28.6
2	7	2.1	38	5.2	15	18.8	60	5.3
3	0	0	8	1.1	3	3.8	11	1.0

*sd* standard deviation, *n* number, *ASC-US* atypical squamous cells of undetermined significance, *LSIL* low-grade squamous intraepithelial lesion, *ASC-H* atypical squamous cells, cannot exclude high-grade squamous intraepithelial lesion, *HSIL* high-grade squamous intraepithelial lesion, *AGC* atypical glandular cells, *AIS* adenocarcinoma in situ, *CIN*, cervical squamous intraepithelial neoplasia

Missing data: a, 105; b, 175; c, 8; d, 75

Social and demographic characteristics, cytological, colposcopic, and histopathological findings of the participants are shown in Table 1. Mean age was 31.0 years, 60.6% of the patients had more than two sexual partners, 53.9% used hormonal contraceptives, most had one or no full-term pregnancies (80.1%) and were never smokers (84.9%). Of the 1138 files, in 741 (65.1%) none of the 3 images were present, in 326 (28.6%) one of them was detected, in 60 (5.3%) 2 of them were identified, and in 11 (1.0%) the 3 images were visualized simultaneously (Table 1).

Table 2 shows the prevalence of the three images in the participants stratified according to their histopathological diagnosis. The prevalence of at least one of the three images distributed according to the histopathological diagnosis was 18% (60/334) for ≤ CIN 1, 37.4% (271/724) for CIN 2 and 3, and 82.5% (66/80) for AIS. Isolatedly, the most frequently identified images were grouped glands (117/724, 16.2%) and aceto-white villi (141/724, 19.5%) in cases of CIN 2 and 3, and aceto-white villi (43/80, 53.8%) and atypical vessels (27/80, 33.8%) in cases of AIS (Table 2).

**Table 2:** Prevalence of three colposcopic images in 1138 participants correlated with histopathological diagnosis

Image	≤ CIN 1 (*n* = 334) *f* (%)	CIN 2 and 3 (*n* = 724) *f* (%)	AIS (*n* = 80) *f* (%)	TOTAL (*n* = 1.138) *f* (%)
Obstructed dilated grouped glands	34 (10.2)	117 (16.2)	17 (21.3)	168 (14.8)
Aceto-white villi with invaginated borders fused or not	16 (4.8)	141 (19.5)	43 (53.8)	200 (17.6)
Atypical vessels in cylindrical epithelium area	17 (5.1)	67 (9.3)	27 (33.8)	111 (9.8)
At least one of the three new images	60 (18.0)	271 (37.4)	66 (82.5)	397 (34.9)

*CIN* cervical squamous intraepithelial neoplasia, *AIS* adenocarcinoma in situ, *n* number, *f* frequency

For the diagnosis of AIS, the sensitivity of the images showing grouped glands, aceto-white villi, and atypical vessels was 21.3% (CI: 17.3–25.2%), 53.8% (CI: 48.9–58.6%), and 33.8% (CI: 29.2–38.3%), respectively, whereas the specificity was 89.8% (CI: 86.9–92.7%), 95.2% (CI: 93.2–97.3%), and 94.9% (CI: 92.8–97.0%), respectively (Table 3). For the diagnosis of CIN 2 and 3, the sensitivity of the images showing grouped glands, aceto-white villi, and atypical vessels was 16.2% (CI: 13.9–18.4%), 19.5% (CI: 17.1–21.9%), and 9.3% (CI: 7.5–11.0%), respectively, while the specificity was 89.8% (CI: 88.0–91.6%), 95.2% (CI: 93.9–96.5%), and 94.9% (CI: 93.6–96.2%), respectively (Table 4).

**Table 3:** Sensitivity, specificity, diagnostic accuracy, and positive likelihood value of three colposcopic images for the diagnosis of adenocarcinoma in situ

Image	AIS		Estimated performance
	Positive *n* (%)	Negative* n* (%)	
Obstructed dilated grouped glands			
Positive	17 (33.3)	34 (66.7)	Sensitivity: 21.3 (17.3–25.2)
			Specificity: 89.8 (86.9–92.7)
Negative	63 (17.4)	300 (82.6)	Accuracy: 76.6 (72.5–80.7)
			LR + : 2.1 (0.7–3.5)
Aceto-white villi with invaginated borders fused or not			
Positive	43 (72.9)	16 (27.1)	Sensitivity: 53.8 (48.9–58.6)
			Specificity: 95.2 (93.2–97.3)
Negative	37 (10.4)	318 (89.6)	Accuracy: 87.2 (84.0–90.4)
			LR + : 11.2 (8.2–14.3)
Atypical vessels in cylindrical epithelium area			
Positive	27 (61.4)	17 (38.6)	Sensitivity: 33.8 (29.2–38.3)
			Specificity: 94.9 (92.8–97.0)
Negative	53 (14.3)	317 (85.7)	Accuracy: 83.1 (79.5–86.7)
			LR + : 6.6 (4.2–9.0)
At least one of the three new images			
Positive	66 (52.4)	60 (47.6)	Sensitivity: 82.5 (78.8–86.2)
			Specificity: 82.0 (78.3–85.7)
Negative	14 (4.9)	274 (95.1)	Accuracy: 82.1 (78.4–85.8)
			LR + : 4.6 (2.6–6.6)

*AIS* adenocarcinoma in situ, *95% CI* 95% confidence interval, *n* number, *LR + * positive likelihood ratio

**Table 4:** Sensitivity, specificity, diagnostic accuracy, and positive likelihood value of three colposcopic images for the diagnosis of cervical squamous intraepithelial neoplasia grades 2 and 3

Images	CIN 2 and 3		Estimated performance % (95% CI)
	Positive *n* (%)	Negative *n* (%)	
Obstructed dilated grouped glands			
Positive	117 (77.5)	34 (22.5)	Sensitivity: 16.2 (13.9–18.4)
			Specificity: 89.8 (88.0–91.6)
Negative	607 (66.9)	300 (33.1)	Accuracy: 39.4 (36.5–42.4)
			LR + : 1.6 (0.8–2.3)
Aceto-white villi with invaginated borders fused or not			
Positive	141 (89.8)	16 (10.2)	Sensitivity: 19.5 (17.1–21.9)
			Specificity: 95.2 (93.9–96.5)
Negative	583 (64.7)	318 (35.3)	Accuracy: 43.4 (40.4–46.4)
			LR + : 4.1 (2.9–5.3)
Atypical vessels in cylindrical epithelium area			
Positive	67 (79.8)	17 (20.2)	Sensitivity: 9.3 (7.5–11.0)
			Specificity: 94.9 (93.6–96.2)
Negative	657 (67.5)	317 (32.5)	Accuracy: 36.3 (33.4–39.2)
			LR + : 1.8 (1.0–2.6)
At least one of the three new images			
Positive	271 (81.9)	60 (18.1)	Sensitivity: 37.4 (34.5–40.3)
			Specificity: 82.0 (79.7–84.3)
Negative	453 (62.3)	274 (37.7)	Accuracy: 51.5 (48.5–54.5)
			LR + : 2.1 (1.2–2.9)

*CIN* cervical squamous intraepithelial neoplasia, *CI* confidence interval, *n* number, *LR + * positive likelihood ratio

The accuracy of the images showing grouped glands, aceto-white villi, and atypical vessels for the diagnosis of AIS was 76.6% (CI: 72.5–80.7%), 87.2% (CI: 84.0–90.4%), and 83.1% (CI: 79.5–86.7%), respectively (Table 3), while for the diagnosis of CIN 2 and 3 it was 39.4% (CI: 36.5–42.4%), 43.4% (CI: 40.4–46.4%), and 36.3% (CI: 33.4–39.2%), respectively (Table 4). Additionally, LR + of grouped glands, aceto-white villi, and atypical vessels for the diagnosis of AIS was 2.1 (CI: 0.7–3.5), 11.2 (CI: 8.2–14.3), and 6.6 (CI: 4.2–9.0), respectively (Table 3), whereas for the diagnosis of CIN 2 and 3, it was 1.6 (CI: 0.8–2.3), 4.1 (CI: 2.9–5.3), and 1.8 (CI: 1.0–2.6), respectively (Table 4).

Considering the presence of at least one of the three images for the diagnosis of AIS, sensitivity, specificity, accuracy, and LR + were 82.5% (CI: 78.8–86.2%), 82.0% (CI: 78.3–85.7%), 82.1% (CI: 78.4–85.8%), and 4.6 (CI: 2.6–6.6), respectively (Table 3), while for the diagnosis of CIN 2 and 3, they were 37.4% (CI: 34.5–40.3%), 82.0% (CI: 79.7–84.3%), 51.5% (CI: 48.5–54.5%), and 2.1 (CI: 1.2–2.9), respectively (Table 4).

**Fig. 3:**
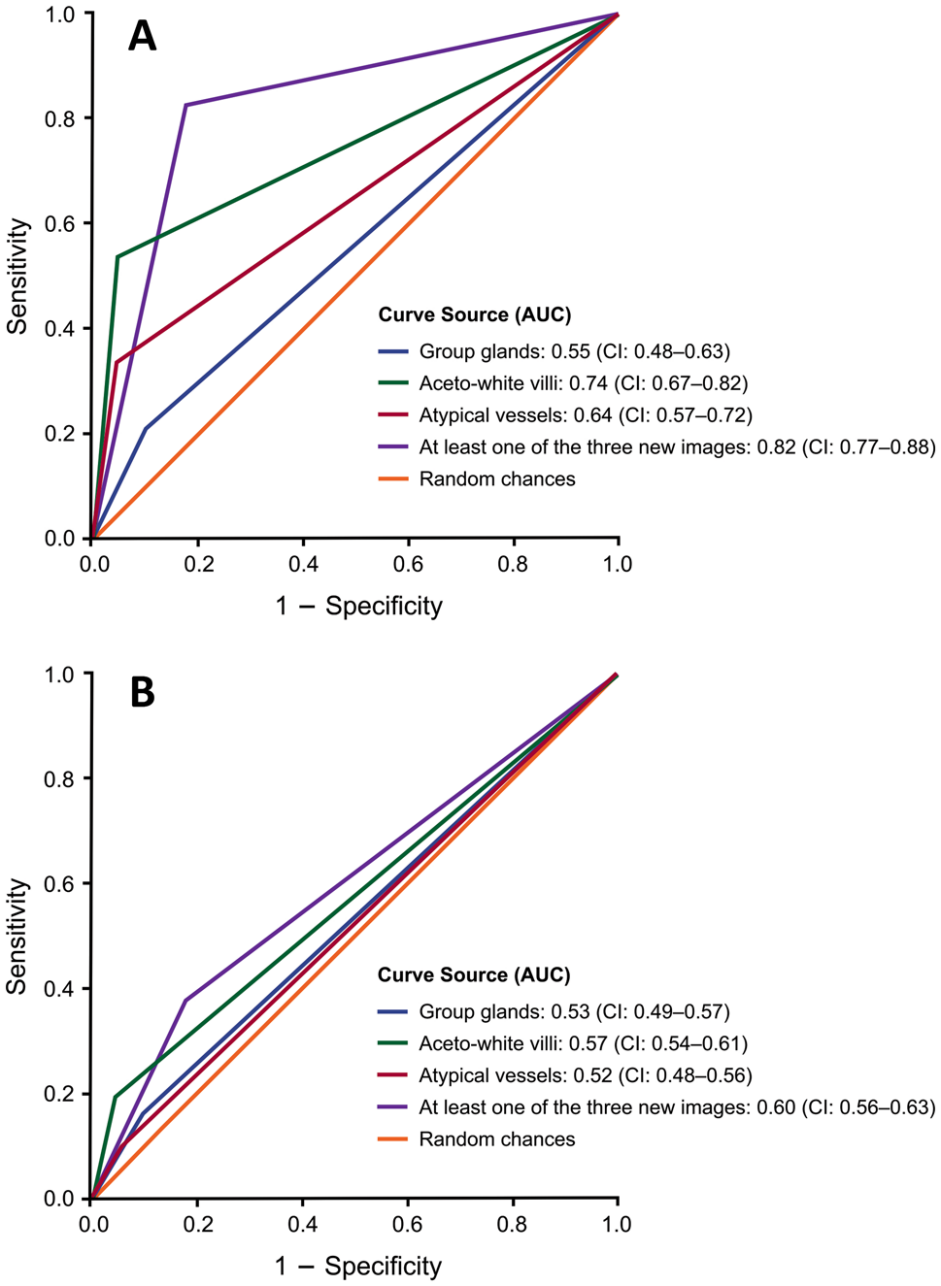
Receiver operating characteristic (ROC) curves resulting from regression analyses shown as solid lines. **a** Sensitivity and 1 – Specificity of the three colposcopic images, alone or associated, for the diagnosis of adenocarcinoma in situ; **b** Sensitivity and 1 – Specificity of the three colposcopic images, alone or associated, for the diagnosis of cervical squamous intraepithelial neoplasias grades 2 and 3

ROC curves showed that AUC of grouped glands, aceto-white villi, and atypical vessels was 0.55 (CI: 0.48–0.63), 0.74 (CI: 0.67–0.82), and 0.64 (CI: 0.57–0.72) for the distinction between AIS and ≤ CIN 1, respectively (Fig. 3a). Moreover, for the distinction between CIN 2 and 3 and ≤ CIN 1, AUC of grouped glands, aceto-white villi, and atypical vessels was 0.53 (CI: 0.49–0.57), 0.57 (CI: 0.54–0.61), and 0.52 (CI: 0.48–0.56) (Fig. 3b). Also, AUC regarding the presence of at least one of the three images for the detection of AIS was 0.82 (CI: 0.77–0.88) (Fig. 3a), whereas for the detection of CIN 2 and 3 it was 0.60 (CI: 0.56–0.63) (Fig. 3b).

## Discussion

It was noteworthy to detect that the three colposcopic images here evaluated were more prevalent among glandular cervical precursor neoplasias than among squamous lesions; moreover, the three images showed higher accuracy for the identification of the former than of the latter. Among the three images, the most prevalent was aceto-white villi followed by grouped glands. In addition, the images showing aceto-white villi and atypical vessels had higher sensitivity, LR + , and AUC for detecting glandular cervical precursor neoplasias than squamous ones.

In a previous study also carried out by our team, which included 1571 participants, the sensitivity of the cytological abnormality ASC-H + and AIS + to identify CIN 2 + [44.0% (95% CI: 41.0–47.0%) and 72.0% (95% CI: 67.0–76.0%), respectively] was lower than the sensitivity for major or suspicious for invasion colposcopy findings to identify CIN 2 + [62.0% (95% CI: 60.0–65.0%) and 86.0% (95% CI: 83.0–89.0%), respectively]. However, the specificity of the former was higher [79.0% (95% CI: 77.0–81.0%) and 79.0% (95% CI: 75.0–83.0%), respectively] compared to the latter [59.0% (95% CI: 57.0–62.0%) and 59.0% (95% CI: 55.0–64.0%)], respectively. The low and lesser specificity of major or suspicious for invasion colposcopy findings reiterates that some sort of screening prior to colposcopy is essential to identify potentially positive patients to precursor neoplasias of cervical cancer [16].

The identification of cytological abnormalities through screening in healthy women indicates the performance of a confirmatory test with higher specificity [17]. Consequently, the ideal use of colposcopy requires better specificity for identifying cervical precursor neoplasias, especially because women referred to colposcopy have already been identified as patients at risk through cytological screening.

During the identification of AIS and CIN 2 and 3, the high specificities of the three images, grouped glands (89.8%, CI: 86.9–92.7% and 89.8%, CI: 88.0–91.6%), aceto-white villi (95.2%, CI: 93.2–97.3% and 95.2%, CI: 93.9–96.5%), and atypical vessels (94.9%, CI: 92.8%–97.0% and 94.9%, CI: 93.6–96.2%), pointed out a low number of false positive results. Therefore, in this study, most patients who presented with any of the three images had a diagnosis compatible with cervical precursor neoplasias, and among these, the glandular type was proportionally the most frequent. In contrast, the sensitivity of the three images for the detection of glandular and squamous cervical precursor neoplasias was low. This means that, in the absence of these images, both types of neoplasias were found with a high frequency (false negative results).

For the detection of glandular cervical precursor neoplasias, the analysis of the presence of at least one of the three images compared to that of each image isolatedly resulted in considerably higher sensitivity (82.5%, CI: 78.8–86.2%) accompanied by a slight reduction in specificity (82.0%, CI: 78.3–85.7%). Also, the detection of squamous cervical precursor neoplasias through the presence of at least one of the three images led to a slight increase in sensitivity (37.4%, CI: 34.5–40.3%) and a subtle loss in specificity (82.0%, CI: 79.7–84.3%) compared to the analysis of each image isolatedly. The high specificity of aceto-white villi and atypical vessels colposcopy findings, their high LR + , and their AUC greater than 0.50, for the diagnosis of AIS, suggest that training colposcopists to recognize these images could lead to an improvement in the colposcopy diagnostic performance for the detection of invasive adenocarcinoma precursor neoplasias.

The high specificity and low sensitivity found in our study are similar to the results of studies that evaluated the performance of two specific images, inner border sign and ridge sign, for the diagnosis of CIN 2 and 3 [18–20]. These images were introduced in the current colposcopy terminology of IFCPC^9^ due to the evidence that they represent relevant signs for the identification of CIN 2 and 3 [19, 21]. However, different than our work, the other studies did not evaluate the performance of the images regarding the diagnoses of each of the main histopathological types of cervical precursor neoplasias, squamous or glandular, since they assessed all the cervical precursor neoplasias together.

In a classic meta-analysis of colposcopy performance, in cases the cutoff point of the colposcopic examination changes from the threshold normal cervix to any types of CIN (1, 2, or 3) or AIS to the threshold ≤ CIN 1 to CIN 2 and 3 or AIS, sensitivity decreases and simultaneously specificity, likelihood ratio, and AUC increase [22]. This emphasizes the need of using the classic major findings for colposcopic evaluation.

The group of 334 patients with diagnosis of ≤ CIN 1 had an unexpected high proportion of major colposcopy findings (34.1%), which could be attributed mostly to the subjectivity of the colposcopy itself. The high sensitivity and low specificity of colposcopy are most likely due to the overcalling of low-grade lesions, which could be attributed to the fact that vascular atypia is the hallmark of higher grades lesions. Yet, vascular atypia can also be the result of HPV infection without intraepithelial lesions [22]. Moreover, among the total cases of major colposcopy findings (785), only 14.5% had their final diagnosis of ≤ CIN 1.

Nevertheless, none of the aforementioned studies [18–20, 22] involved assessing colposcopy performance for the diagnosis of glandular cervical precursor neoplasias. Conversely, they all added up to several other studies to demonstrate the high specificity of colposcopy [9, 18–20, 23], in line with the findings of this study.

Given the nonexistence of studies on colposcopy performance for the diagnosis of glandular cervical precursor neoplasias, it is of paramount importance to conduct researches to provide evidences in this field. Moreover, the rarity of this type of neoplasia and the existence of colposcopic mimics such as squamous metaplasia, condylomas, invasive adenocarcinoma, and microglandular hyperplasia, make it difficult for colposcopists to acquire experience in their clinical practice [10].

The likelihood ratio is a useful tool to assess how good a diagnostic test is, especially because it is less likely to change with the prevalence of the disorder than sensitivity, specificity, and predictive values [24]. This propriety is particularly suitable to this study, inasmuch as it compares colposcopy findings in neoplasias with high (CIN 2 and 3) and low (AIS) prevalence. In our study, the likelihood ratio showed a clear demarcation, albeit with slight variations, between diagnoses of glandular and squamous cervical precursor neoplasias for aceto-white villi (11.2 and 4.1) and atypical vessels (6.6 and 1.8) images.

In addition, AUC indicated a reasonable colposcopy performance (0.74; CI: 0.67–0.82) based on the presence of aceto-white villi images for the diagnostic forecast of AIS or the absence of a precursor neoplasia (≤ CIN 1). Nevertheless, for squamous cervical precursor neoplasias, none of the three images exhibited sufficient diagnostic performance, and the values of their AUC were comparable to those obtained by chance [25]. Finally, the results here obtained for AUC and LR + indicate a better performance of aceto-white villi images to AIS diagnosis and its possibility of being a helpful tool in the distinction between glandular and squamous cervical precursor neoplasias.

The changes introduced in the cytological classification and screening for detecting high risk HPV [5] aimed to improve the sensitivity in screening programs. Nonetheless, an increase in sensitivity leads to a decrease in specificity [26]. Moreover, as a result of HPV vaccination, in situations of high coverage, the lesions screened are likely to be more subtle [11]. This emphasizes the importance of multiple biopsies [27–29] or biopsies of any images reacting to acetic acid [30]. Thereupon colposcopy performance should be considered an evolving process. Furthermore, both the description and evaluation of image patterns, in our study and in previous ones [18–20], are relevant for achieving the goal of improving specificity of colposcopy.

Among the limitations of this study, we should mention: the reviewer did not indicate the biopsy placement and knew the referral cytology; analysis of static images, since colposcopy involves longitudinal assessment of changes caused by acetic acid. However, it has already been evidenced that the interpretation of static images does not significantly differ from that of images corresponding to cervical precursor neoplasias in real time [31]. The advantages of our study are: size of the sample; high number of glandular cervical precursor neoplasias included, considering their relative rarity; inclusion of colposcopic images with visible SCJ; gold standard of diagnosis represented by histopathological examination of the specimen obtained by conization; use of ROC curve and likelihood ratio to evaluate the performance of the three images, since these parameters, unlike the predictive values, are not influenced by the prevalence of disease in the studied sample [24].

## Conclusions

This study showed that the prevalence and accuracy of the three images evaluated were higher for the diagnosis of glandular cervical precursor neoplasias compared to squamous lesions. Sensitivity, LR + , and AUC of the images showing aceto-white villi and atypical vessels were higher for detecting glandular precursor neoplasias than squamous ones. These results suggest that colposcopists training on the detection of these images could lead to improvements on the colposcopic performance for invasive adenocarcinoma precursor neoplasias. Conducting further studies is still necessary to support these findings and extend the research.

## Data Availability

The dataset supporting the conclusions of this article is included within the article.
